# Disseminated Fusobacterium necrophorum Infection Without Jugular Venous Thrombophlebitis: An Atypical Presentation of Lemierre's Syndrome

**DOI:** 10.7759/cureus.87700

**Published:** 2025-07-10

**Authors:** Yoshitaka Tomoda, Naoki Wasai, Kohei Ukai, Hiroaki Tanaka, Satoshi Kitaura

**Affiliations:** 1 General Internal Medicine, Itabashi Chuo Medical Center, Itabashi, JPN; 2 Infectious Disease, Itabashi Chuo Medical Center, Itabashi, JPN

**Keywords:** atypical lemierre syndrome, fusobacterium necrophorum, hepatic vein thrombosis, liver abscess, pulmonary empyema, septic pulmonary emboli

## Abstract

Lemierre's syndrome is classically characterized by *Fusobacterium necrophorum* bacteremia with jugular venous thrombophlebitis, typically affecting young adults following oropharyngeal infections. We report an unusual case of disseminated *F. necrophorum* infection in a 53-year-old man who presented without pharyngitis or jugular venous thrombophlebitis, instead manifesting with septic pulmonary emboli, bilateral empyema, liver abscess, and portal vein thrombophlebitis. This case highlights the importance of recognizing atypical presentations of invasive *Fusobacterium* infections, which are associated with higher mortality rates compared to classic Lemierre's syndrome. Early diagnosis and aggressive multimodal treatment led to the successful management of this complex case.

## Introduction

*Fusobacterium necrophorum* is an anaerobic gram-negative bacillus commonly found in the oropharyngeal flora [1]. *F.* *necrophorum* bacteremia is traditionally associated with internal jugular vein thrombophlebitis in young adults-a condition known as Lemierre's syndrome. In Lemierre’s syndrome, secondary thrombophlebitis of the internal jugular vein develops subsequent to primary oropharyngeal infection, predominantly affecting young individuals, with consequent septic embolization to various anatomical sites [2]. However, recent literature suggests that approximately 30% of *F. necrophorum* bacteremia cases occur without neck involvement, particularly in older patients with comorbidities including malignancy [3]. These atypical presentations are often misdiagnosed initially and carry significantly higher mortality rates [3]. Furthermore, emerging reports have described abdominal variants of Lemierre's syndrome, in which *Fusobacterium* species cause visceral abscesses and septic thromboses involving not only the portal vein but also the hepatic vein [4,5]. These presentations complicate the identification of the primary infectious focus and pose diagnostic and therapeutic challenges distinct from classical Lemierre’s syndrome. We present a case of extensive *Fusobacterium* infection without jugular venous thrombophlebitis, emphasizing the importance of recognizing this increasingly reported abdominal variant of invasive *Fusobacterium* disease.

## Case presentation

A 53-year-old male plumber with a 35-pack-year smoking history and long-term alcohol consumption (approximately 14 beers weekly for over 30 years) presented with a six-day history of fever and nausea, recently complicated by exertional back pain. Notably, he denied any preceding sore throat or neck pain. On admission, vital signs showed temperature 38.7°C, blood pressure 132/71 mmHg, heart rate 130 beats/minute, respiratory rate 20 breaths/minute, and oxygen saturation 96% on room air. Physical examination revealed conjunctival icterus and bilateral basilar crackles on lung auscultation. There was no evidence of pharyngeal erythema, dental caries, cervical lymphadenopathy, or neck stiffness. Laboratory studies demonstrated leukocytosis (34,400/μL), severe thrombocytopenia (5,000/μL), elevated C-reactive protein (16.3 mg/dL), renal impairment (serum creatinine 2.02 mg/dL), and hyperbilirubinemia (total bilirubin 3.9 IU/L) (Table 1).

**Table 1:** Initial laboratory findings on admission.

Test	Result	Normal range	Units
WBC (white blood cells)	34,400	3,300–8,600	μL
Hemoglobin	14.2	13.7–16.8	g/dL
Platelet	5,000	158,000–348,000	μL
Serum creatinine	2.0	0.61–1.08	mg/dL
Blood urea nitrogen	56.6	8–23	mg/dL
Total bilirubin	3.9	0.3–1.2	IU/L
AST (aspartate aminotransferase)	52	8–40	IU/L
ALT (alanine aminotransferase)	35	5–45	IU/L
C-reactive protein	163	0–1.4	mg/L

**Figure 1:**
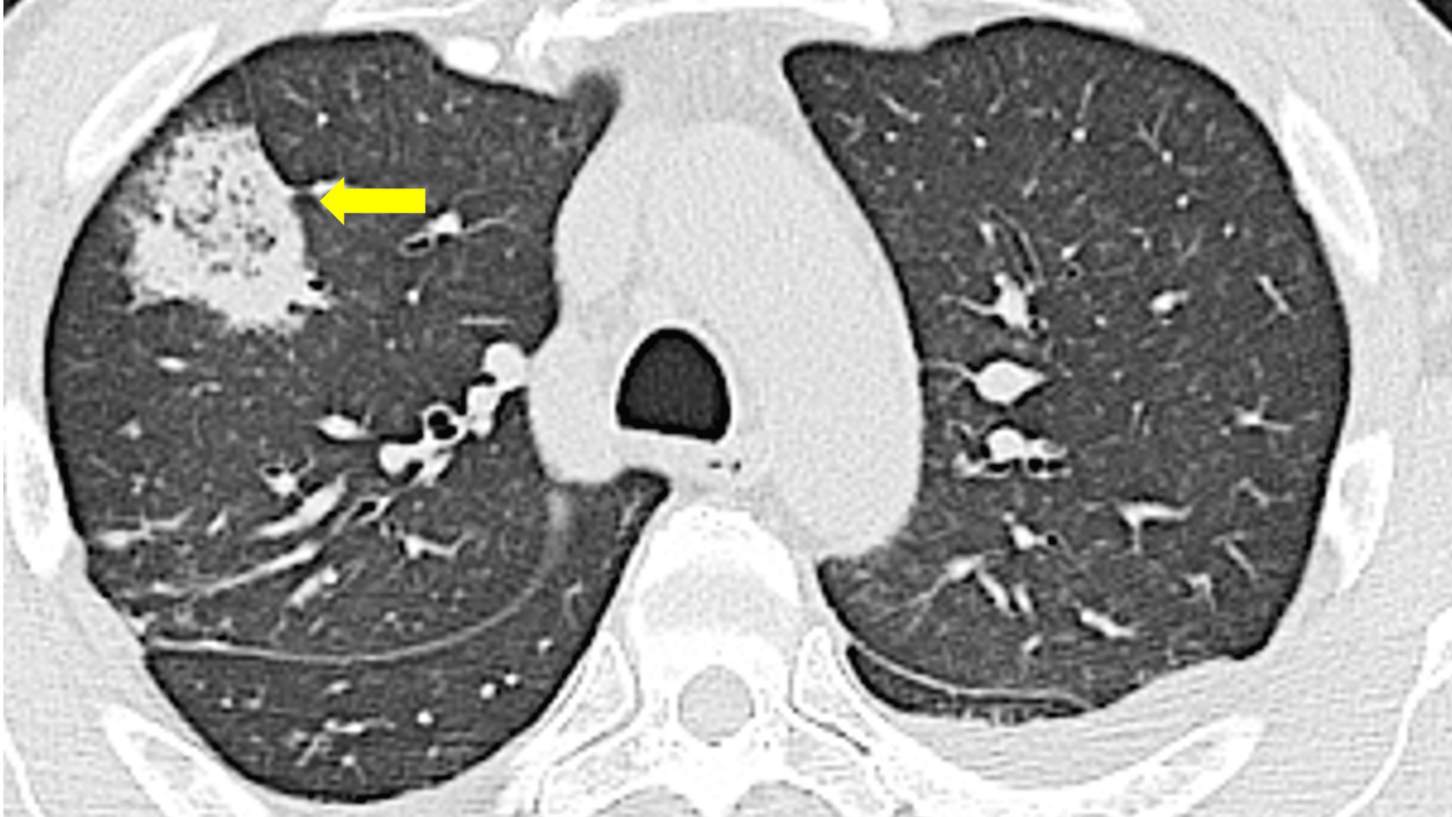
Chest computed tomography (CT) image on hospital day 1 demonstrating a mass with reversed halo sign in the right upper lobe of the lung (yellow arrow).

**Figure 2:**
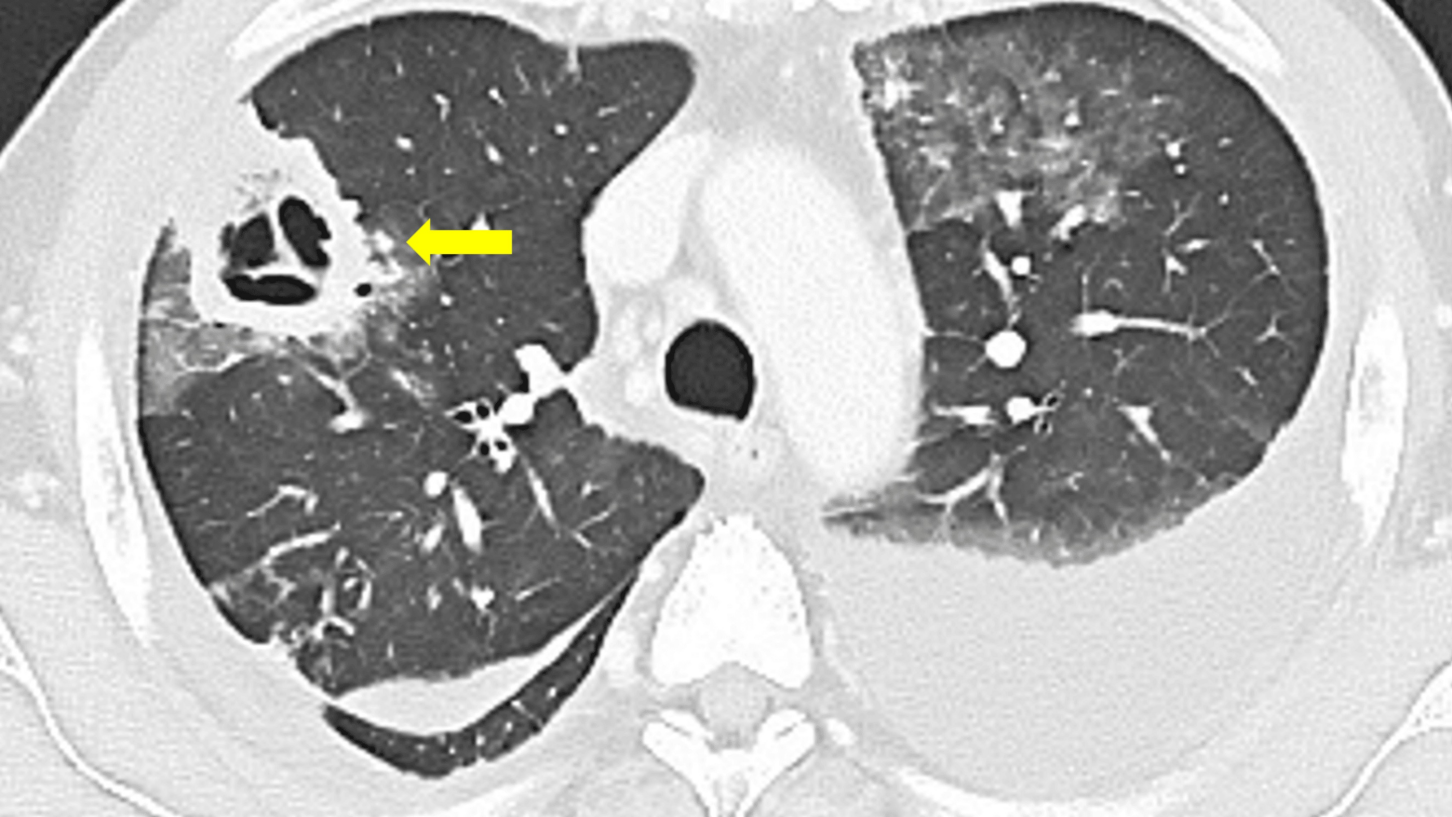
Chest computed tomography (CT) on hospital day 3 demonstrating cavitary change in the mass and bilateral pleural effusion (yellow arrow).

**Figure 3:**
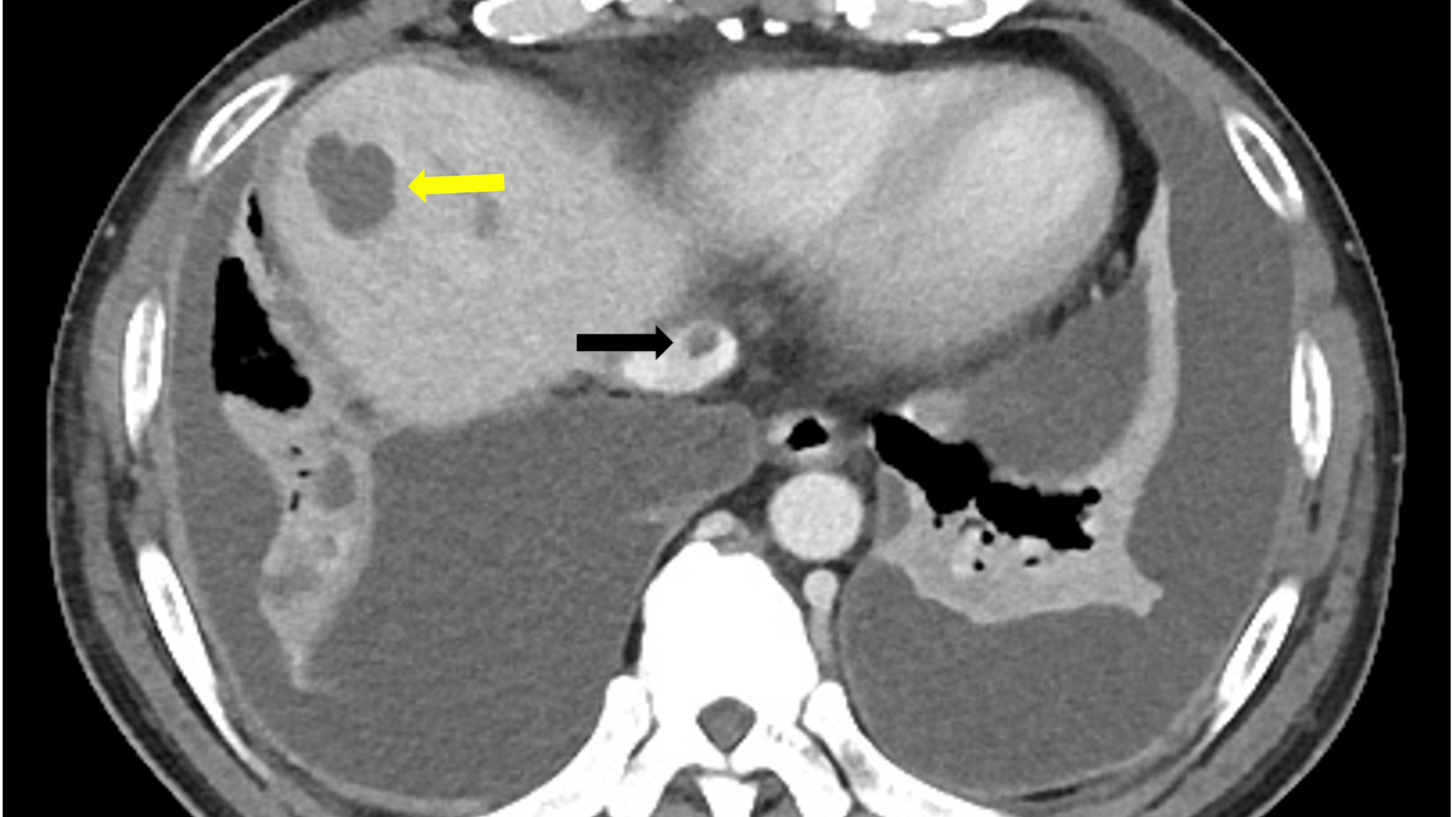
Contrast-enhanced abdominal computed tomography (CT) revealed liver abscess (yellow arrow) and inferior vena cava thrombosis (black arrow).

**Figure 4:**
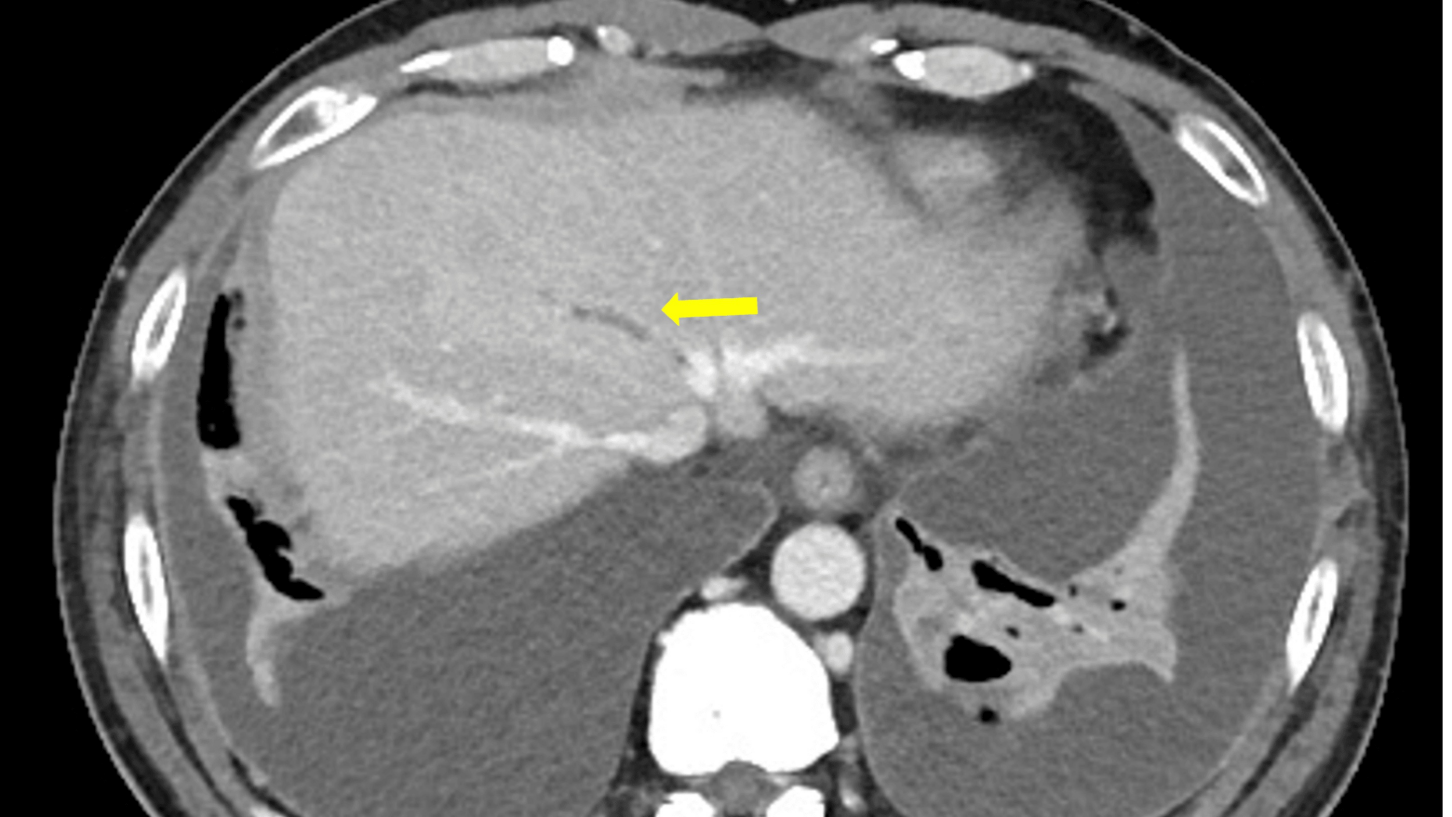
Contrast-enhanced abdominal computed tomography (CT) revealed hepatic vein thrombosis (yellow arrow).

**Figure 5:**
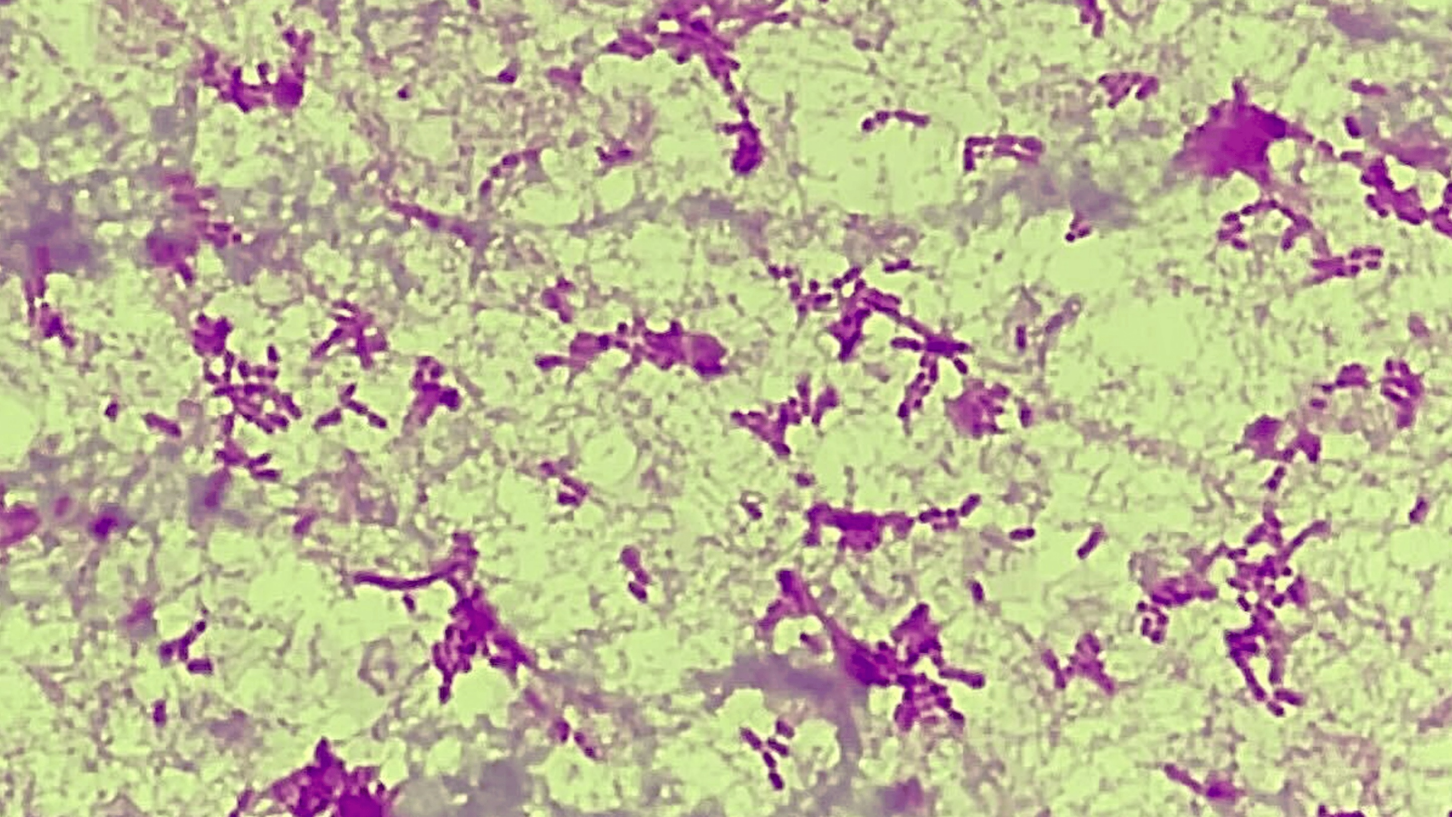
Gram staining of the peripheral blood demonstrating gram-negative bacilli.

Peripheral blood smear showed no schistocytes. Initial chest and abdominal computed tomography (CT) revealed multiple pulmonary nodules displaying a characteristic reversed halo sign and a low-density liver lesion (Figure 1). Despite empiric ceftriaxone therapy, the patient's respiratory status deteriorated. A follow-up contrast-enhanced CT on hospital day three demonstrated progression of pulmonary lesions with cavitation development, increasing bilateral pleural effusion, hepatic vein thrombosis, inferior vena cava (IVC) thrombosis, and a definitive liver abscess (Figures 2-4). Importantly, meticulous examination of neck vessels confirmed the absence of internal jugular vein thrombosis. Blood cultures drawn on admission yielded *F. necrophorum* on hospital day four, with notable hemolysis of the culture medium (Figure 5). Thoracentesis produced purulent fluid, though subsequent cultures remained negative. Transthoracic echocardiography showed no valvular vegetation.

We diagnosed disseminated *F. necrophorum* infection manifesting as septic pulmonary emboli, bilateral empyema, liver abscess, and septic portal vein thrombophlebitis-an atypical presentation of Lemierre's syndrome without the hallmark jugular venous involvement. Treatment was intensified by switching antibiotics to ampicillin/sulbactam and implementing aggressive surgical interventions: bilateral thoracic drainage, video-assisted thoracic surgery (VATS) for empyema decortication, and percutaneous drainage of the liver abscess. Extensive evaluation including gastroscopy and colonoscopy revealed no obvious primary source of infection. The patient improved gradually and was discharged after 34 days of hospitalization on oral metronidazole to complete a four-week antibiotic course. No relapse has occurred during follow-up.

## Discussion

This case represents a diagnostically challenging abdominal variant of Lemierre's syndrome. While the classical presentation involves young adults with recent pharyngitis and internal jugular vein thrombophlebitis, our patient demonstrated several atypical features: older age, absence of oropharyngeal symptoms, lack of jugular venous thrombosis, and the presence of hepatic vein and IVC thromboses in association with a liver abscess and bilateral empyema. A single case report describing the simultaneous occurrence of all three complications (lung abscess, empyema, and hepatic venous system thrombosis) in the absence of internal jugular vein thrombosis has not been clearly documented in the identified literature.

The primary pathogens of the *Fusobacterium* genus are *F. necrophorum* and *Fusobacterium nucleatum*. These bacteria are more pathogenic than most of the typical anaerobic flora and are linked to fast-progressing infections, such as peritonsillar abscesses, diverticulitis, liver abscesses, and bacteremia [6]. *Fusobacterium* bacteremia is infrequent, with past incidence rates reported between 1.5 and 3.7 cases per million annually, though recent years have shown an upward trend [7].

Traditional diagnostic criteria for Lemierre's syndrome include (1) recent oropharyngeal infection, (2) metastatic lesions, and (3) internal jugular vein thrombophlebitis or *Fusobacterium* bacteremia [8]. However, growing evidence suggests that invasive *Fusobacterium* infections without neck involvement constitute an important clinical entity that affects primarily older patients with underlying comorbidities [9,10]. These atypical cases have significantly higher mortality rates (approximately 14%) compared to classical Lemierre's syndrome (2%), possibly due to delayed diagnosis and treatment [3].

Liver abscesses and septic thrombosis of the hepatic venous system are extremely rare complications of *Fusobacterium* infections. While portal vein thrombosis has been described in abdominal variants of Lemierre's syndrome, hepatic vein and especially IVC thrombosis are far less common. In the literature, only a few prior cases of hepatic vein thrombosis associated with *F. nucleatum* bacteremia have been reported [5], and to our knowledge, *Fusobacterium*-associated IVC thrombosis has not been previously documented. The presence of both hepatic vein and IVC thromboses in our case suggests an aggressive pattern of intravascular dissemination.

Typically, pyogenic liver abscesses are secondary to biliary tract infections or portal vein seeding from intra-abdominal sources such as appendicitis or diverticulitis. However, in Lemierre's syndrome and its variants, hematogenous dissemination from oropharyngeal or other mucosal sites is postulated to be the primary mechanism [8]. In a recent review of *Fusobacterium*-associated liver abscesses, approximately one-third of cases were considered cryptogenic, with no identifiable intra-abdominal or biliary source, and *F. nucleatum* was found to be an equally common causative agent as *F. necrophorum* [11]. This aligns with our case, where exhaustive evaluation-including upper and lower gastrointestinal endoscopy-failed to reveal any primary intra-abdominal or biliary tract focus. These findings support the hypothesis that the liver abscess in our patient likely resulted from hematogenous seeding as part of a disseminated *Fusobacterium* infection. Furthermore, the associated hepatic vein and IVC thromboses suggest a particularly aggressive intravascular course, consistent with previously reported abdominal variants of Lemierre's syndrome [9,10].

The unique radiological findings in our case merit discussion. The reversed halo sign-a central ground-glass opacity surrounded by consolidation-was initially observed in our patient's pulmonary lesions. While once considered pathognomonic for cryptogenic organizing pneumonia, this sign occurs in approximately 59% of septic pulmonary emboli and can be a valuable early diagnostic clue [12,13]. The rapid evolution to cavitation on subsequent imaging is consistent with the aggressive nature of *Fusobacterium* infections.

The bilaterality of empyema further distinguishes this case, as empyema complicates only about 10% of Lemierre's syndrome cases and is usually unilateral [6,14]. Bilateral empyema is more severe than unilateral cases and poses additional challenges in treatment. Thoracic drainage on both sides can restrict patient mobility and increase the likelihood of postoperative complications. Furthermore, one-lung ventilation under anesthesia can be challenging in these cases due to impaired oxygenation [15]. In instances where medical treatment fails, surgical intervention, such as simultaneous bilateral VATS decortication, has been shown to be effective for treating bilateral empyema [16]. Management of such complex cases requires a multidisciplinary approach combining appropriate antimicrobial therapy targeting anaerobic organisms with timely and sometimes multiple surgical interventions. The simultaneous bilateral VATS decortication performed in our patient exemplifies the aggressive surgical management sometimes necessary in these severe infections.

## Conclusions

This case underscores an increasingly recognized abdominal variant of Lemierre's syndrome, characterized by disseminated *F. nucleatum* infection without internal jugular venous thrombophlebitis. Despite the absence of oropharyngeal symptoms and cervical vein thrombosis, the patient developed severe metastatic complications including septic pulmonary emboli, bilateral empyema, hepatic abscess, and concurrent hepatic vein and IVC thromboses. These findings illustrate the potential for *Fusobacterium* species to cause aggressive intravascular dissemination beyond the classical head and neck territory. Clinicians should maintain a high index of suspicion for incomplete or abdominal variants of Lemierre's syndrome in patients-particularly older adults-presenting with anaerobic bacteremia and visceral abscesses, even when oropharyngeal or biliary sources are not evident. Early recognition, initiation of appropriate anaerobic antimicrobial therapy, and timely surgical intervention, such as bilateral VATS decortication when indicated, are essential to achieving favorable outcomes in these life-threatening infections.
